# Analysis of multiple phenotypes in genome-wide genetic mapping studies

**DOI:** 10.1186/1471-2105-14-151

**Published:** 2013-05-02

**Authors:** Chen Suo, Timothea Toulopoulou, Elvira Bramon, Muriel Walshe, Marco Picchioni, Robin Murray, Jurg Ott

**Affiliations:** 1 Key Laboratory of Mental Health, Institute of Psychology, Chinese Academy of Sciences, Beijing, 100101, China; 2 Department of Medical Epidemiology and Biostatistics, Karolinska Institutet, Stockholm, Sweden; 3 Department of Psychology, The University of Hong Kong, Hong Kong, Hong Kong; 4 State Key Laboratory of Brain and Cognitive Sciences, The University of Hong Kong, Hong Kong, Hong Kong; 5 King's College London, King's Health Partners, Department of Psychosis Studies Institute of Psychiatry, London, UK; 6 Institute of Psychiatry, King’s College, London, UK; 7 St Andrew’s Academic Centre, Kings College London, Northampton, UK; 8 St Andrew’s Academic Centre, Kings College London, Northampton, UK

**Keywords:** Multiple phenotypes, Statistical method, Genetic mapping

## Abstract

**Background:**

Complex traits may be defined by a range of different criteria. It would result in a loss of information to perform analyses simply on the basis of a final clinical dichotomized affected / unaffected variable.

**Results:**

We assess the performance of four alternative approaches for the analysis of multiple phenotypes in genetic association studies. We describe the four methods in detail and discuss their relative theoretical merits and disadvantages. Using simulation we demonstrate that PCA provides the greatest power when applied to both correlated phenotypes and with large numbers of phenotypes. The multivariate approach had low type I error only with independent phenotypes or small numbers of phenotypes. In this study, our application of the four methods to schizophrenia data provides converging evidence of the relative performance of the methods.

**Conclusions:**

Via power analysis of simulated data and testing of experimental data, we conclude that PCA, creating one variable based on a linear combination of all the traits, performs optimally. We propose that our comparison will provide insight into the properties of the methods and help researchers to choose appropriate strategy in future experimental studies.

## Background

For linkage and genetic association studies of biological markers, a complex trait can be defined by a range of multiple and often overlapping criteria. For example, obesity, usually defined by body mass index (BMI), is also related to waist-hip ratio (WHR) and body fat percentage. More than one indicator is usually used. It is possible that the specific type of the indicator selected may favor one susceptibility gene, while selection on another indicator may reveal another gene. In early genome-wide association studies, a common variant of the FTO gene was implicated in increased BMI and to predispose to childhood and adult obesity [1]. Later, a meta-analysis of 61 studies concluded that multiple loci affect WHR independently of BMI [2]. In this example, WHR and BMI may reflect different aspects of the underlying gene effect, demonstrating the importance of utilizing multiple phenotype data in the analysis, although chance fluctuation may also lead to the different results of BMI and WHR.

Multiple intermediate phenotypes have been proposed for a variety of neuropsychiatric disorders, in particular schizophrenia, bipolar disorder, and Alzheimer’s disease. For example in Alzheimer’s disease, impairment occurs in eight cognitive domains including attention, language, memory, perceptual skills, constructive abilities, orientation, problem solving and functional abilities [3]. Typically the resulting measures are statistically or functionally correlated, which then increases the difficulty of handling such multivariate data. So when subjects clinically are diagnosed as either affected or unaffected for a disorder, this dichotomization may lead to a loss of power in genetic analyses.

In a recent review, Ott *et al.*[4] described four approaches to tackle multiple phenotypes. The first is the most general and proposes to analyze each phenotype individually and correct for multiple testing by the Bonferroni method. The second is similar to the first but proposes permutation analysis to address problems of multiple testing. The third is to treat different phenotypes as a multivariate regression problem. The last is to transform all phenotype data into a single overall phenotype using principal component analysis (PCA) and then to perform standard univariate regression at each biomarker. There is so far no consensus on which method is the best.

The primary purpose of our study was to assess the performance of these four approaches. We introduce the four methods in detail and discuss their relative advantages and disadvantages. Then through power analysis of extensively simulated data and a real data application, we conclude that for genetic association studies, using PCA to create one variable based on a linear combination of all the traits performs optimally.

## Methods

### One at a time

The most intuitive and simplest way to deal with multiple phenotypes is to test each SNP against one phenotype at a time. In the case of quantitative traits, a one-way analysis of variance (ANOVA) is usually performed. It tests whether the mean of a phenotype is the same in the three genotypes, AA, AB, and BB. As an alternative to ANOVA, we can perform a simple linear regression for each phenotype as a response variable and the genotypes as predictors (this analysis has 1 degree of freedom [df] versus 2 for ANOVA). Each of the phenotypes would measure a trait from a particular different angle. A given SNP could be associated with none, one or more of the phenotypes. In practice, researchers have to make decisions on the criteria for declaring significance and interpretation. Usually, if any of the phenotypes result in a statistically significant outcome, the SNP is retained for further investigation. This is also what we do in this study, that is, if the smallest *p*-value across phenotypes is less than a pre-determined threshold, we suspect the SNP is a genetic risk factor for the trait.

Since multiple tests are conducted at a SNP, we need to handle the resulting *p*-values by controlling the overall type I error. In the situation of a single test, a result is declared significant when *p* ≤ 0.05 if the type I error α is controlled at 5% by convention. With *m* independent tests, the probability of making correct decisions on all the results is (1 – *p*)^m^, given the null hypothesis is true. So the probability of finding at least one false positive is 1 – (1 – *p*)^m^. This overall type I error α is called the family-wise error rate. It is approximated by *mp* when *m* is large and *p* is small and we want to keep α ≤ 0.05 [5]. Thus, we should set 0.05/*m* as the threshold to declare a single test significant. This approach represents the well-known Bonferroni correction. Unfortunately, the disadvantage is that the correction tends to be too stringent when tests are dependent, which is often the case with endophenotypes. This correction causes more false negatives so power is decreased. An alternative to Bonferroni correction is to use permuted p-values as discussed in the next section.

### Permuted p-values

For a given SNP, we define the best test statistic, *F*_max_ or *P*_min_, among associations with all phenotypes as our final test statistic for this SNP (*P*_min_ is preferable in the presence of a mixture of categorical and quantitative phenotypes). In the first approach of one SNP at a time, we assess the significance level associated with a test statistic by looking it up in a known statistical distribution table. Since the null distribution of *P*_min_ may not have a known distribution, we approximate it by simulating datasets under no association. The procedures are firstly to permute sample labels in phenotypes but keeping genotypes in their original order. Obviously, in this new dataset we destroy any association between phenotypes and genotypes by randomization. Secondly, we obtain and store the smallest *p*-value in such a dataset. We repeat the randomization a sufficiently large number of times. The smallest p-values stored would approximate the distribution of *P*_min_ under the null hypothesis. Finally, we calculate the proportion of the smallest p-values in the distribution less than or equal to the observed *P*_min_ to be the significance level associated with *P*_min_[6]. It is often believed that a permuted *p*-value is not as conservative as a Bonferroni-corrected p-value and, thus, is more powerful. We will revisit this issue in the simulation section.

### Multivariate analysis

The obvious drawback to the first two approaches is that they do not utilize information from the structure of multiple phenotypes, which may or may not to be correlated. Given there are not too many phenotypes, we could carry out regression or multivariate analysis of variance (MANOVA) with multiple phenotypes directly. In multivariate regression, the response variables are assumed to follow some specific multivariate distribution, most commonly a multi-normal distribution, although this is a strong and sometimes unwarranted assumption.

### Principal component analysis

Another method of analyzing several phenotypes simultaneously is to summarize them into one overall value. The simplest summary statistic is to take the mean or sum (often called a "scale"). But in real-life examples, directly adding phenotype values does not always make sense. For example, it is difficult to interpret one's height plus weight plus intelligence quotient (IQ). Instead of a simple sum, we may consider a weighted linear combination of phenotypes with weights based on the inverse of their variances. In principal component analysis (PCA), such a weighted sum is called the first principal component (PC). This technique of dimension reduction is often used in the presence of a large number of predictors in a regression model. Here, we apply the technique to condense information in outcome variables, that is, phenotypes.

### Software

All the simulation and data analysis described in this paper was conducted in the R statistical programming language (http://www.r-project.org).

## Dataset and preprocessing procedures

We first describe our analysis of a dataset on schizophrenia, followed by extensive simulation of computer-generated data. Investigators collected clinical, cognitive, MRI and genetic information of European subjects in families with schizophrenia and controls. Family members include mainly parents and siblings. All case subjects are individuals with a diagnosis of schizophrenia. Additional description of the studies, including methods for selecting control subjects and diagnosis is provided in Toulopoulou *et al*., (2003; 2003S; 2004; 2007; 2010) [7-11] and Owens *et al*., [12] and in the supplementary material (see Additional file 1).

## Results

### Real data analysis

We select all available 216 unrelated schizophrenia patients and 240 unrelated controls and analyze genotypes in case and control subjects for each of 51 candidate SNPs potentially associated with schizophrenia. Phenotypes of interest are subjects’ cognition level measured from the angle of IQ and memory. More explicitly, they are two summary scores from the Wechsler Adult Intelligence Scale (WAIS) test to measure intelligence through verbal and performance subtests, one score from the National Adult Reading Test (NART) estimating premorbid intelligence levels, and two scores from the Wechsler Memory Scale (WMS) test that measures logical memory in the form of immediate memory capacity and delayed memory performance. We apply the four approaches described in the methodology section to conduct two sets of analyses: one with all five phenotypes and the other with the three IQ phenotypes only.

Results are given in Table 1, where we see that the three IQ and two memory variables are correlated with an average correlation coefficient of 0.59. Complete phenotype data are available for a total of 118 case subjects. Rates of missing genotypes ranged from 21% to 56%. Out of 51 SNPs, rs3738401 is ranked highest (smallest *p*-value) regardless of which method is used. It has previously been identified to be associated with schizophrenia in the Scottish population with a relative risk of 5 [13]. Note that in our data, this SNP is associated with the five phenotypes (three IQ and two memory variables) only in patients and not in controls. Among the four methods, PCA results in the smallest *p*-value of 0.002. The one-by-one and permutation approaches have similar p-values of 0.008 and 0.009, respectively, followed by MANOVA with a *p*-value of 0.031.

**Table 1:** Top five SNPs discovered using the four methods

**Five phenotypes: 3 IQ + 2 memory measurement;**				$\bar{r}$ **= 0.59;**	***n* = 118**
One-by-one*	rs3738401	rs9822602	Dys1578kb	rs2005976	rs821616
	0.009	0.042	0.074	0.078	0.208
Permutation	rs3738401	rs9822602	Dys1578kb	rs2005976	rs821616
	0.008	0.028	0.049	0.052	0.131
MANOVA	rs3738401	rs9822602	Dys1325kb	rs5105774	rs2604578
	0.031	0.046	0.048	0.097	0.126
PCA	rs3738401	rs5105774	rs9822602	Deletion_A7_Ms	rs720309
	0.002	0.056	0.063	0.121	0.139
Five phenotypes: 3 IQ measurement;				$\bar{r}$ = 0.72;	*n* = 131
One-by-one*	rs3738401	rs9822602	rs2005976	rs5751229	rs1347003
	0.037	0.04	0.084	0.103	0.149
Permutation	rs3738401	rs9822602	rs2005976	rs5751229	rs1347003
	0.029	0.031	0.061	0.068	0.101
MANOVA	Dys1325kb	rs2005976	rs9822602	rs3738401	rs5751229
	0.016	0.046	0.066	0.093	0.245
PCA	rs3738401	rs5751229	rs9822602	Deletion_A7_Ms	rs2005976
	0.015	0.095	0.099	0.106	0.122

*Bonferroni correction is applied to correct for multiple testing among phenotypes. The exact formula used is 1 - (1 - *p*)^m^.

There are two sets of analyses, one with five moderately correlated phenotypes and the other with three highly correlated phenotypes.

When we reduce the phenotypes to a highly correlated subset, the three IQ scores have a correlation coefficient of 0.72. We notice that MANOVA is then no longer able to pick up the potential risk variant, rs3738401, at a significance level of 0.05. The significance of this SNP in the other three methods also drops, possibly due to higher correlation, or fewer phenotypes, or both, although the sample size is slightly increased. We will investigate the relationship between power and correlation coefficient, number of phenotypes and so on in the next section.

### Simulation

We simulate extensively to assess which of the approaches would give highest power under various settings. We assume a multivariate, normally distributed phenotype associated with a risk SNP. Frequency of allele A is set to 0.3 throughout the simulation study. Other parameters in the simulation are the number of individuals *n*, the number of phenotypes *m*, effect size *δ*, and the correlation coefficient *r* among the phenotypes. Here, *δ* means that if we set the mean of phenotypes for individuals with genotype BB to be unit 1, mean of phenotypes for individuals with genotype AB and AA would be equal to *δ* and *δ*^*2*^, respectively. This inheritance model is analogous to the genotypic relative risk model, in which the chance of an individual having the disease increases by a factor *δ* with an increasing copy of the risk allele in the genotypes [14]. We vary values of these parameters to investigate the pattern of power for the four approaches.

Firstly, we examine the relationship between power and correlation among the phenotypes. While the parameters *n*, *m* and *δ* are fixed at 300, 10 and 1.2, respectively, *r* varies from 0 to 0.9, that is, from complete independence to a high correlation. We want to generate 10,000 replicates of the SNP of interest, that is, 20,000 of its alleles. To generate a genotype, we assume Hardy Weinberg equilibrium (HWE) so that the two alleles in a genotype can be ascertained independently. To achieve this, allele A is generated from a binomial distribution (10,000, 0.3), and separately, allele B from a binomial distribution (10,000, 0.7). Under the assumption of HWE, given an allele frequency of 0.3, the mean genotype frequencies of AA, AB and BB are 0.09, 0.42 and 0.49, respectively. Phenotype data are then generated from a multivariate normal distribution with mean values of *δ*^*2*^, *δ* and 1.

**Figure 1:**
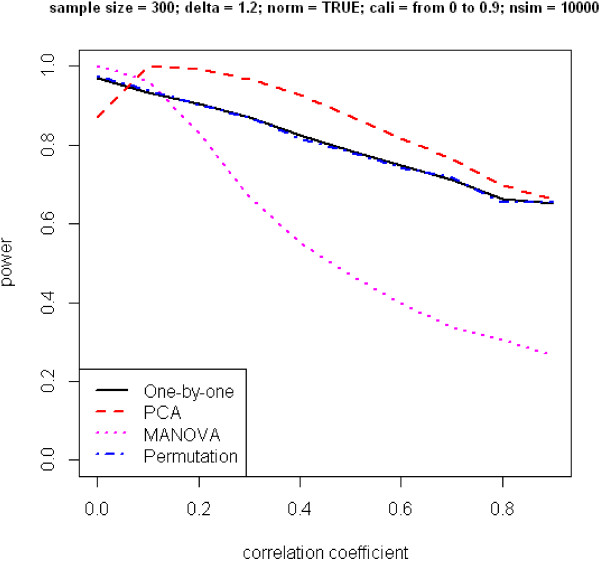
**Power versus correlation coefficient.** Phenotypes are generated from multivariate normal distribution. Correlation coefficient ranges from 0 to 1.

**Figure 2:**
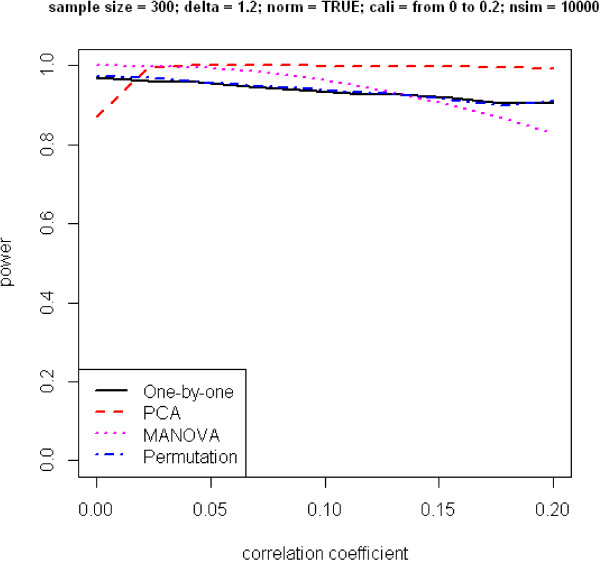
**Power versus correlation coefficient.** Phenotypes are generated from multivariate normal distribution. Correlation coefficient ranges from 0 to 0.2.

In Figure 1, power is plotted against the correlation coefficient under the alternative hypothesis of *δ* ≠ 1. The patterns for the change of power are quite different for the four approaches. When phenotypes are correlated, even to a mild degree, MANOVA performs the worst. Its power decreases dramatically with an increase of the correlation coefficient. Performance of the one-by-one and permuted *p*-value approaches also has an inverse relationship with the correlation coefficient, although their rates of decrease are slower. The PCA approach has the most interesting pattern, which does not show as a monotonic curve. Power first increases, then decreases. To take a closer look at where the peak occurs, we simulate another set of SNPs with all parameter settings the same as above except the correlation coefficient varies from 0 to 0.2. Figure 2 reveals the maximum power to occur when the correlation coefficient is approximately 0.05. We are not sure how to explain this unusual pattern.

**Figure 3:**
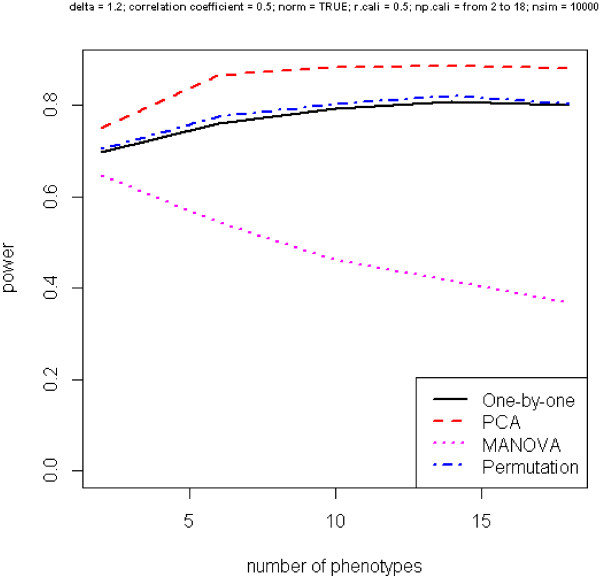
**Power versus number of phenotypes.** Phenotypes are moderately correlated.

Next, we check the relationship between power and number of phenotypes, as shown in Figure 3, where *r* is fixed at 0.5. Interestingly, for all methods except MANOVA, power increases with more phenotypes included. The performance of the one-by-one and permuted p-value approaches goes hand in hand and PCA is the best.

**Figure 4:**
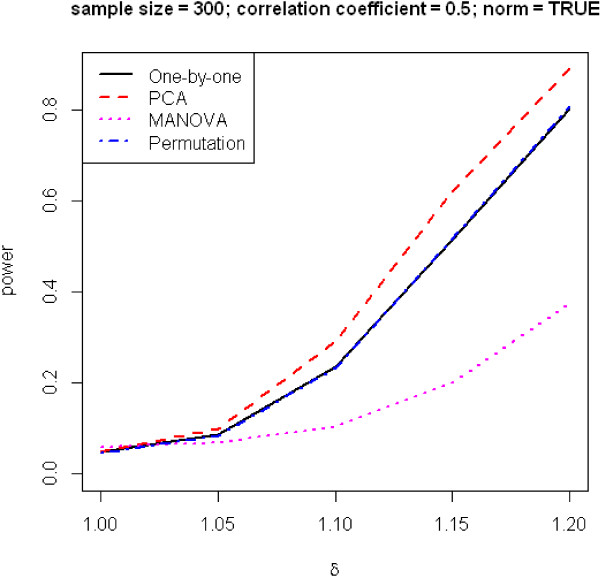
**Power versus effect size.** Number of phenotypes *m* = 10.

**Figure 5:**
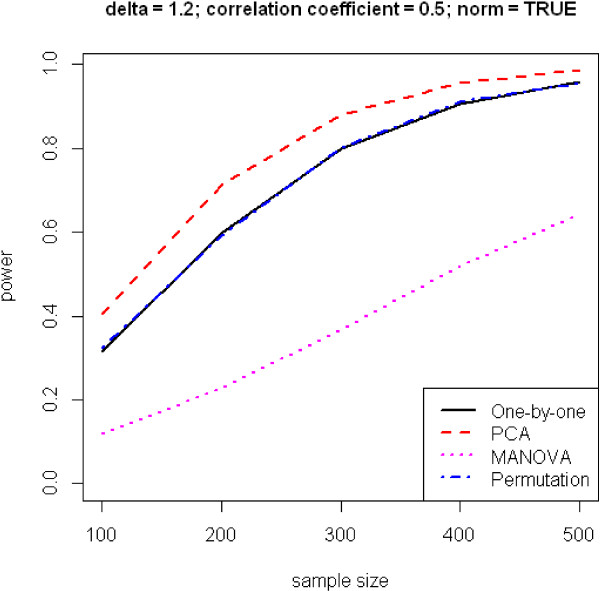
**Power versus sample size.** Number of phenotypes *m* = 10.

In the end, we present two traditional graphs in power analysis, where power is plotted against effect size and sample size, shown in Figures 4 and 5. As expected, power improves with increases of sample size and effect size. PCA has again the best performance, followed by the one-by-one and permutation methods, which do not really differ, and MANOVA.

Note that for all simulations above, power is calculated as the proportion of *p*-values less than a pre-determined threshold. In practice, we often fix 0.05 as that threshold such that the estimated type I error is controlled at about 5% and, in principle, power is expected to be about 5% under the null hypothesis, H_0_: δ = 1. However, we do not know whether it is always the same case for the four approaches, especially when correlation coefficient and number of phenotypes also vary. In supplementary method in Additional file 1, we address the issue of selecting a proper threshold in detail.

We also want to check the performance of these four methods when phenotype distributions deviate from normal. Using the same parameter setting as above, we find similar patterns for the four methods (See supplementary Figures 2 and 3 in Additional file 1).

## Discussion

We have described four methods to analyze multiple observed phenotypes for linkage and association studies of complex traits. For any given marker, it is likely that a simple dichotomized phenotype of affected versus unaffected is not clearly associated with the marker due to relatively subjective definition and characterization of disease status, especially for psychiatric traits. Disease definition is not generally based on the genetics of the trait but is established for the purpose of unique classification. Thus, a range of phenotypes is required to capture all underlying genetic risk factors with different functions. Hypertension in Lyon hypertensive rats is a good example. It was shown that two different blood pressure measurements, diastolic and pulse, are associated with different genes which might have been missed based on the conventional measurement of systolic and diastolic blood pressure [15]. Furthermore, because of the correlation structure of many of the phenotypes measured, using PCA to combine the attributes linearly to then simultaneously analyze all phenotypes may be more informative than a straightforward univariate or multivariate approach. Our results from both real and simulated data imply that statistical power and validity can be increased through the PCA approach.

In this study, we investigate the performance of different approaches to analyse multiple continuous phenotypes and recommend PCA as the optimal method. When phenotypes comprise both discrete and continuous variables, each discrete phenotype can be non-linearly transformed [16] before being included as input of the PCA. It is worth studying explicitly the performance of different approaches on both continuous and categorical data in future work. In addition, PCA provides several components. In Additional File 1, we further discuss whether it is better to include more PCs as outcome variables. Preliminary results show that PCA with the first two PCs does not achieve the same power as PCA with the first PC, but still performs among the best especially when correlation between phenotypes is relatively low (see supplementary Figure 4 in Additional file 1).

When phenotype distributions deviate from normality, we check the performance of the four methods and again conclude that PCA is optimal. Although a transformation of the non-normality, e.g. using Box-Cox or simply log transformations, would improve power to be similar to that for normally distributed variables, we show through simulation that the consequence would be decrease in power if we did not remove non-normality in our phenotype distribution. Comparing Figure 1 and Supplementary Figure 2, PCA appears to be less sensitive to the assumption of normality.

In practice it is not uncommon to have missing values, which may differently affect the four methods. The univariate approaches may be less affected in the sense that if a missing value exists in one of the phenotypes, we are still able to select a minimal p-value among the other phenotypes. But the standard PCA and MANOVA require that all values be present for the phenotypes. Fortunately, there are methods available to get around the problem of missing data, for example imputation. So we can predict and fill in missing values before implementing any method to analyse the multi-phenotype data. Additionally, an R package, pcaMethods, allows performing PCA on incomplete data and may be used for missing values estimation [17].

Working with the different methods brought several issues to our attention. We found that using Bonferroni correction and permuted p-value performed comparably in terms of power in the two univariate approaches. At first glance, this result seems surprising, given that the Bonferroni correction is known to be conservative when phenotypes are strongly correlated. However, it is worth noting that we use a calibrated threshold throughout such that power is 5% under the null hypothesis and, thus, we have a fair comparison in terms of power. As shown in the left panel of Supplementary Figure 1, had we applied a fixed threshold 0.05, the one-by-one approach using Bonferroni correction would be associated with less power than the permuted p-value approach. However, this perceived power difference is not real as it was due to different type 1 error rates when the null hypothesis is true. When considering the burden of computation time in permutation testing and its relatively poor performance compared with PCA in most of the model settings, we conclude that neither of the two univariate methods performs optimally.

## Conclusions

Using simulation we demonstrated that PCA provides the greatest power when applied to both correlated phenotypes and large numbers of phenotypes. The multivariate approach had low type I error only with independent phenotypes or small numbers of phenotypes. Despite increasing awareness of how to deal with multiple phenotypes, the one-by-one approach is still commonly employed by researchers. Examples of using the other methods in real data application are not often seen. In this study, our application of the four methods to schizophrenia data provides converging evidence of the relative performance of the methods. We propose that our comparison will provide some insight into the properties of the methods and help researchers to choose appropriate strategy in future experimental studies.

## Competing interests

The authors declare that they have no competing interest.

## Authors' contributions

JO conceived, designed, directed and helped to draft the manuscript. CS performed the statistical analysis, participated in the design of the study and drafted the manuscript. TT participated in the coordination of the study and helped to draft the manuscript. MP helped to draft the manuscript. TT, MP, EB, MW and RM contributed samples. All authors read and approved the final manuscript.

## Supplementary Material

Additional file 1 **Supplementary material and method.** A PDF containing description in detail of schizophrenia data used for testing the four methods, methodology addressing the issue of selecting a proper threshold to declare significance, and results of the performance of the four methods when phenotype distributions deviate from normal. Click here for file

## References

[B1] Frayling TM Timpson NJ Weedon MN Zeggini E Freathy RM Lindgren CM Perry JR Elliott KS Lango H Rayner NW Shields B Harries LW Barrett JC Ellard S Groves CJ Knight B Patch AM Ness AR Ebrahim S Lawlor DA Ring SM Ben-Shlomo Y Jarvelin MR Sovio U Bennett AJ Melzer D Ferrucci L Loos RJ Barroso I Wareham NJ A common variant in the FTO gene is associated with body mass index and predisposes to childhood and adult obesity Science 2007 316 5826 889 894 Epub 2007/04/17 10.1126/science.1141634 17434869 PMC2646098

[B2] Heid IM Jackson AU Randall JC Winkler TW Qi L Steinthorsdottir V Thorleifsson G Zillikens MC Speliotes EK Mägi R Workalemahu T White CC Bouatia-Naji N Harris TB Berndt SI Ingelsson E Willer CJ Weedon MN Luan J Vedantam S Esko T Kilpeläinen TO Kutalik Z Li S Monda KL Dixon AL Holmes CC Kaplan LM Liang L Min JL Meta-analysis identifies 13 new loci associated with waist-hip ratio and reveals sexual dimorphism in the genetic basis of fat distribution Nat Genet 2010 42 11 949 960 Epub 2010/10/12 10.1038/ng.685 20935629 PMC3000924

[B3] Dubois B Feldman HH Jacova C Dekosky ST Barberger-Gateau P Cummings J Delacourte A Galasko D Gauthier S Jicha G Meguro K O'brien J Pasquier F Robert P Rossor M Salloway S Stern Y Visser PJ Scheltens P Research criteria for the diagnosis of Alzheimer's disease: revising the NINCDS-ADRDA criteria Lancet Neurol 2007 6 8 734 746 Epub 2007/07/10 10.1016/S1474-4422(07)70178-3 17616482

[B4] Ott J Wang J Multiple phenotypes in genome-wide genetic mapping studies Protein Cell 2011 2 7 519 522 10.1007/s13238-011-1059-5 21647556 PMC4875235

[B5] Zhang Q Ott J Lin S, Zhao H Multiple Comparisons/Testing Issues Handbook on Analyzing Human Genetic Data: Computational Approaches and Software 2009 Berlin: Springer 277 287

[B6] Manly BFJ Randomization, bootstrap, and Monte Carlo methods in biology 2007 3 Boca Raton, FL: Chapman & Hall/ CRC 455

[B7] Toulopoulou T Morris RG Rabe-Hesketh S Murray RM Selectivity of verbal memory deficit in schizophrenic patients and their relatives Am J Med Genet B Neuropsychiatr Genet 2003 116B 1 1 7 10.1002/ajmg.b.10027 12497605

[B8] Toulopoulou T Rabe-Hesketh S King H Murray RM Morris RG Episodic memory in schizophrenic patients and their relatives Schizophr Res 2003 63 3 261 271 10.1016/S0920-9964(02)00324-9 12957705

[B9] Toulopoulou T Grech A Morris RG Schulze K McDonald C Chapple B Rabe-Hesketh S Murray RM The relationship between volumetric brain changes and cognitive function: A family study on schizophrenia Biol Psychiatry 2004 56 6 447 453 10.1016/j.biopsych.2004.06.026 15364043

[B10] Toulopoulou T Picchioni M Rijsdijk F Hua-Hall M Ettinger U Sham P Murray R Substantial Genetic Overlap Between Neurocognition And Schizophrenia: Genetic Modeling In Twin Samples Arch Gen Psychiatry 2007 64 12 1348 1355 10.1001/archpsyc.64.12.1348 18056542

[B11] Toulopoulou T Goldberg TE Mesa IR Picchioni M Rijsdijk F Stahl D Stahl D Cherny SS Sham P Faraone SV Tsuang M Weinberger DR Seidman LJ Murray RM Impaired Intellect and Memory A Missing Link Between Genetic Risk and Schizophrenia? Arch Gen Psychiatry 2010 67 9 905 913 10.1001/archgenpsychiatry.2010.99 20819984

[B12] Owen SF Picchioni MM Ettinger U McDonald C Walshe M Schmechtig A Murray RM Rijsdijk F Toulopoulou T Prefrontal deviations in function but not volume are putative endophenotypes for schizophrenia Brain 2012 135 2231 2244 10.1093/brain/aws138 22693145 PMC3381723

[B13] Zhang F Sarginson J Crombie C Walker N St Clair D Shaw D Genetic association between schizophrenia and the DISC1 gene in the Scottish population Am J Med Genet B Neuropsychiatr Genet 2006 141B 2 155 159 Epub 2006/01/04 10.1002/ajmg.b.30274 16389590

[B14] Risch N Merikangas K The future of genetic studies of complex human diseases Science 1996 273 5281 1516 1517 10.1126/science.273.5281.1516 8801636

[B15] Dubay C Vincent M Samani NJ Hilbert P Kaiser MA Beressi JP Kotelevtsev Y Beckmann JS Soubrier F Sassard J Lathrop GM Genetic determinants of diastolic and pulse pressure map to different loci in Lyon hypertensive rats Nat Genet 1993, Apr 3 4 354 357 10.1038/ng0493-354 7981757

[B16] de Leeuw J Patrick M Gifi Methods for optimal scaling in R: The Package homals Journal of Statistical Software 2009 31 4

[B17] Roweis S EM algorithms for PCA and SPCA Neural Information Processing Systems 1998 10 626 632

