# Supplementary material for: Analysis of multiple phenotypes in genome-wide genetic mapping studies
Source: BMC Bioinformatics. 2013 May 2;14:151. doi: 10.1186/1471-2105-14-151 (PMC3655878; doi:10.1186/1471-2105-14-151)
Supplement: Additional file 1 — Supplementary material and method. A PDF containing description in detail of schizophrenia data used for testing the four methods, methodology addressing the issue of selecting a proper threshold to declare significance, and results of the performance of the four methods when phenotype distributions deviate from normal. [file 1471-2105-14-151-S1.pdf]

Supplementary material

The sample comprised families and twins who participated in the Maudsley Family and Maudsley Twin studies of schizophrenia. Families and twins were ascertained by referrals of psychiatric clinics and voluntary care organizations across the United Kingdom. In addition, twins were also recruited from a UK based volunteer twin registry. Recruitment letters for referral of patients with schizophrenia were sent to all consultant psychiatrists working in psychiatric hospitals throughout the UK and to all major voluntary care organizations and charitable bodies. Recruitment of a family was done in two ways, either by approaching the patients themselves first and then asking permission to contact their healthy relatives or by approaching a healthy member of the family first. This was determined mainly by how a patient or a family became known to us i.e. whether it was through a psychiatrist or through a voluntary care organization. Controls were recruited via newspaper advertisements and from a volunteer twin register. Families had at least one member with a diagnosis of schizophrenia or schizoaffective disorder. Twins included both monozygotic and dizygotic twin pairs varying in their concordance for Diagnostic and Statistical Manual of Mental Disorders 4th edition (DSM-IV) schizophrenia or schizoaffective disorder, as well as healthy control twins. Exclusion criteria for all participants were head trauma resulting in loss of consciousness for longer than 5 minutes, alcohol or illicit drug dependence in the previous 12 months and organic brain disorder. An additional exclusion criterion for relatives only was presence of psychosis that did not meet DSM-IV criteria for schizophrenia or schizoaffective disorder. Controls had no personal or family history of psychotic illness, schizophrenia or schizoaffective disorder; however, a history of other axis 1 psychiatric disorders was not an exclusion criterion either for unaffected relatives or the controls.

The studies were approved by the local or multicenter ethics committees and all participants gave written informed consent before participation.

Supplementary method

As clearly shown in left panel of Supplementary Figure 1, the power of one-by-one approach is much smaller than 5% if a fixed threshold has been applied. In this case, power of the four approaches does not correspond under H_0. Good performance of a method could be due to its high power at baseline, or vice versa. So we should calibrate an individual threshold for the four approaches respectively, in order to guarantee a fair comparison. To achieve this, we simulate 10,000 SNP replicates under H_0. It would lead to several significant p-values just by chance. If we control type I error to 0.05, 5% of these 10,000 null SNPs would be declared statistically significant given H_0. So the 5th percentile of the resulting p-values should be used as the cutoff value to declare significance for a given approach. If another 10,000 SNP replicates are simulated under null hypothesis, using the threshold value determined above we have a power of about 5%, as shown in Supplementary Figure 1 on the right. Therefore, it is crucial to calibrate an individual threshold for different method. By doing so, we find that actually permutated *p*-value does not outperform Bonferroni corrected *p*-value used in the one-by-one method.

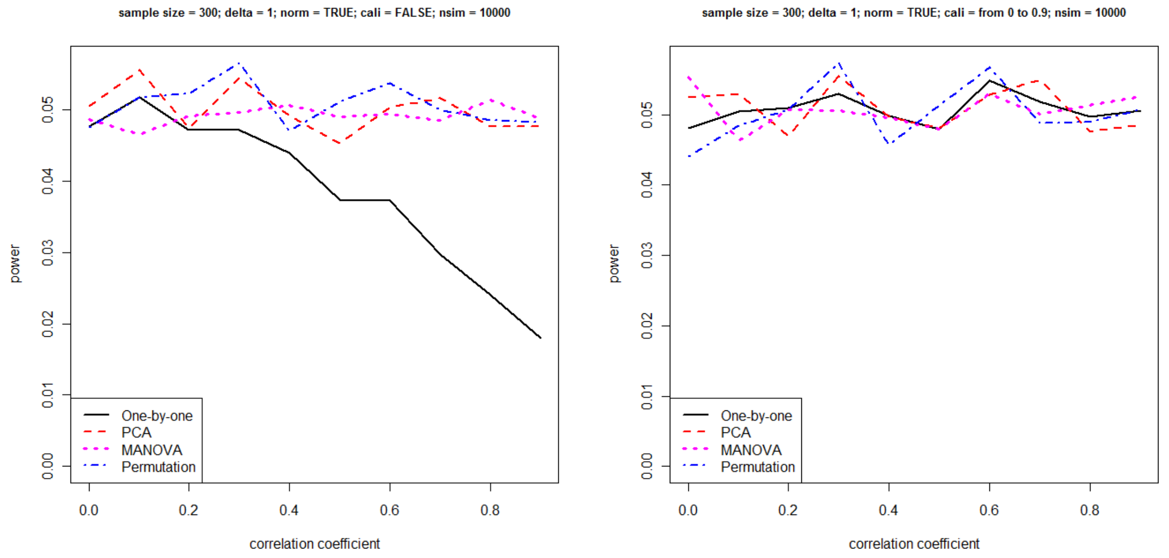

Supplementary Figure 1 - Power versus correlation coefficient

Threshold is set at 0.05 (left) and threshold is calibrated at each *r* such that power is about 5% under null hypothesis (right).

Supplementary figures 2 and 3 present the performance of these four methods when phenotype distributions deviate from normal. Overall, not unexpectedly, power is lower, meaning that we need to increase sample size or we can only identify risk variants with larger effect sizes, if we want to achieve the same power as when phenotypes are normally distributed.

In supplementary Figure 2, the upper panel shows power versus correlation coefficient where the cutoff values are calibrated at individual *r*. On the contrast, the lower panel is when the cutoff values are only calibrated at $r = 0$. Plots in the 1st and 2nd columns are under the hypothesis of $\delta = 1.2$ and 1, respectively. The difference between plots in 2nd and 3rd columns is the scale of y-axis, one from 0 to about 0.05 another from 0 to 1.

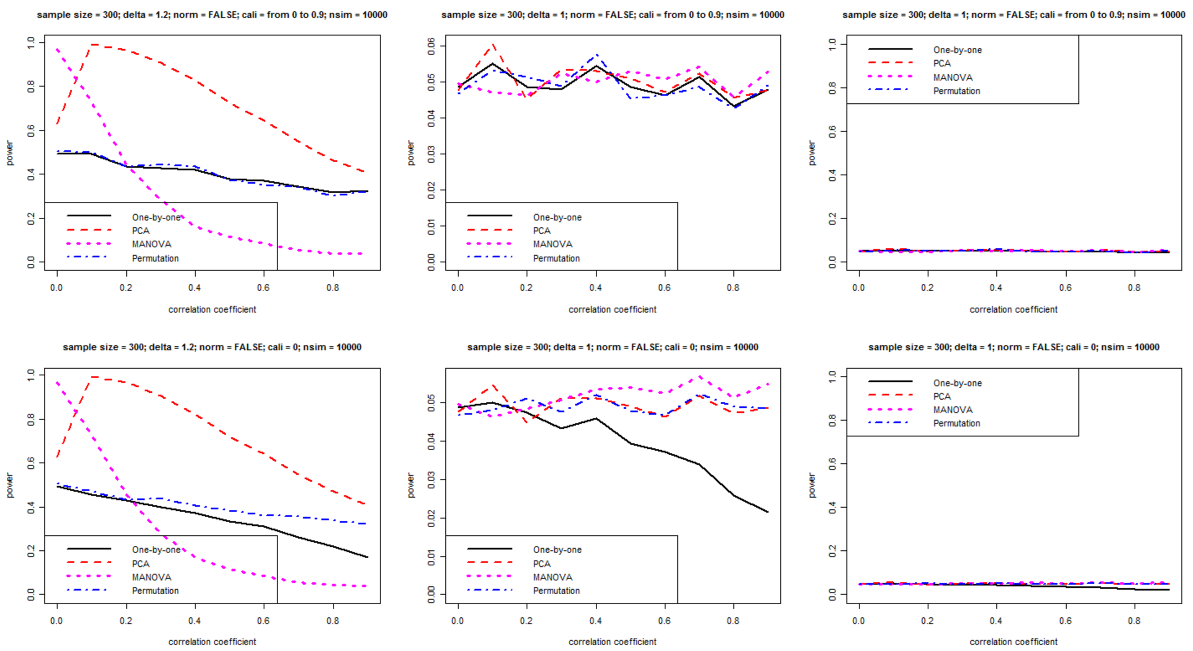

Supplementary Figure 2 - Power versus correlation coefficient

Phenotypes are simulated from log-normal distribution.

In supplementary Figure 3, the upper panel are plots under the hypothesis of $\delta = 1.2$ while lower panel is when $\delta = 1$. The 1st column shows power versus number of phenotypes, where the cutoff values are correctly calibrated at $r = 0.5$ and each number of phenotypes. In the 2nd column, calibration is correctly carried out at each number of phenotypes but at $r = 0$ which is wrong. In the 3rd column, a fixed threshold 0.05 is used.

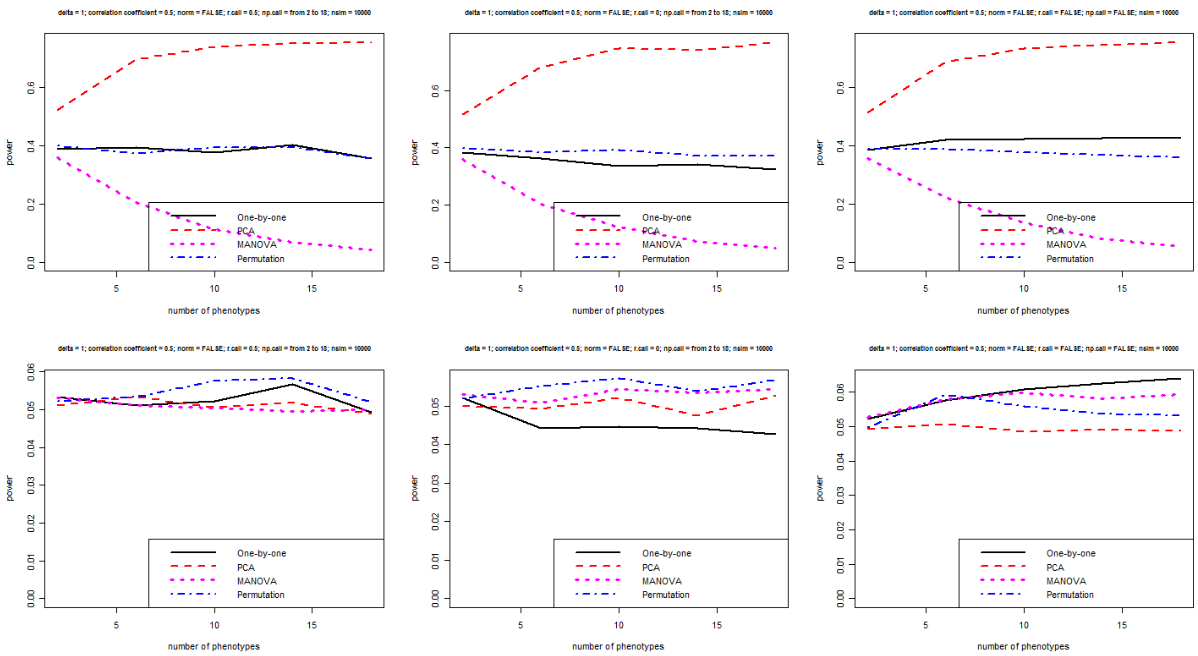

Supplementary Figure 3 - Power versus number of phenotypes

Phenotypes are simulated from log-normal distribution.

PCA can provide several components. To check whether there might be possible power gain by inclusion of additional components, we take PCA with two components as part of the comparison. Under the current simulation scheme, we find that PCA with the first two components does not achieve the same power as PCA with the first PC, but still outperform the other methods as long as correlation between outcome variables are not highly correlated. In practice, we encourage the users to do so by further examine the screen plot, a plot of the eigenvalues. Note that this would depend on the nature of the data and should be accessed case-by-case.

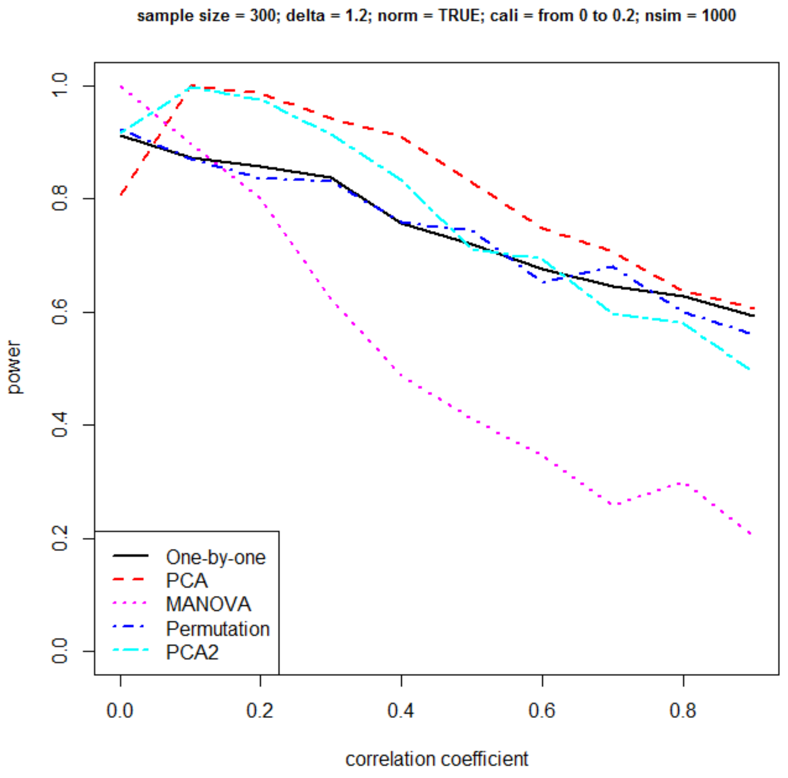

Supplementary Figure 4 - Power versus correlation coefficient.
Phenotypes are simulated from normal distribution. Parameter settings are almost the same as in Figure 1 except that we include one more method in the comparison, PCA with two components. Number of simulated SNPs is limited to 1,000 in order to reduce the computational burden.
